# Assessing, Pricing and Funding Point-of-Care Diagnostic Tests for Community-Acquired Acute Respiratory Tract Infections–Overview of Policies Applied in 17 European Countries

**DOI:** 10.3390/antibiotics11080987

**Published:** 2022-07-22

**Authors:** Sabine Vogler, Friederike Windisch

**Affiliations:** 1 WHO Collaborating Centre for Pharmaceutical Pricing and Reimbursement Policies, Pharmacoeconomics Department, Gesundheit Österreich GmbH (Austrian National Public Health Institute/GÖG), 1010 Vienna, Austria; friederike.windisch@goeg.at; 2 Department of Health Care Management, Technische Universität Berlin, 10623 Berlin, Germany; 3 Department of Management, Institute for Public Management and Governance, Vienna University of Economics and Business, 1020 Vienna, Austria

**Keywords:** antimicrobial drug resistance, antibiotic, respiratory tract infection, policy, diagnostic equipment, health technology assessment, pricing, reimbursement mechanisms, remuneration

## Abstract

Point-of-care diagnostic tests for community-acquired acute respiratory tract infections (CA-ARTI) can support doctors by improving antibiotic prescribing. However, little is known about health technology assessment (HTA), pricing and funding policies for CA-ARTI diagnostics. Thus, this study investigated these policies for this group of devices applied in the outpatient setting in Europe. Experts from competent authority responded to a questionnaire in Q4/2020. Information is available for 17 countries. Studied countries do not base their pricing and funding decision for CA-ARTI diagnostics on an HTA. While a few countries impose price regulation for some publicly funded medical devices, the prices of CA-ARTI diagnostics are not directly regulated in any of the surveyed countries. Indirect price regulation through public procurement is applied in some countries. Reimbursement lists of medical devices eligible for public funding exist in several European countries, and in some countries these lists include CA-ARTI diagnostics. In a few countries, the public payer funds the health professional for performing the service of conducting the test. Given low levels of regulation and few incentives, the study findings suggest room for strengthening pricing and funding policies of CA-ARTI diagnostics to contribute to increased acceptance and use of these point-of-care tests.

## 1. Introduction

Overprescribing and inappropriate prescribing of antibiotics is a major cause of antimicrobial resistance (AMR), which poses a public health threat. In light of the global health and also economic burden of AMR, point-of-care (POC) diagnostic tests can make an important contribution to improved quality of antibiotic prescribing, as they allow identifying and targeting those patients who actually need antibiotics [1,2]. While the value of POC tests has long been known for respiratory diseases [3,4,5], their tremendous benefit from a clinical and public health perspective was confirmed in the COVID-19 pandemic (e.g., use of polymerase chain reaction (PCR) tests) [6,7,8,9].

Empirical studies have proven the impact of POC diagnostic tests, such as C-Reactive Protein (CRP) testing, on reduced, or delayed, antibiotic prescribing [10,11,12,13,14,15], though some uncertainty around the robustness of the results was reported [16]. Thus, POC diagnostic tests constitute one important component in a holistic approach to tackle AMR. Further key policies include AMR stewardship programmes, which comprise action to promote responsible use of antimicrobials and incentives for the development and market launch of novel antibiotics with a lower probability of producing AMR [17,18,19].

Despite being considered as “game-changers” [20], there is room for increased use of the POC diagnostic tests. Their price may be one reason for their limited uptake, since the tests are not always cost-effective compared to antibiotic prescribing [21,22]. In response, it has been proposed to assess the (economic) impact of POC testing from a broader health system perspective based on a longer-term perspective than the one usually taken into account in standard economic evaluations [23].

POC tests inform the health professionals when they decide whether or not an antibiotic shall be prescribed. Therefore, these tests can be considered as companion diagnostics. In European legislation, a companion diagnostic is defined as a device that is essential for the safe and effective use of a corresponding medicine, to identify, before or during treatment, patients who are most likely to benefit from the corresponding medicine or likely to be at increased risk of serious adverse reactions as a result of treatment with the medicine [24]. Usually, companion diagnostics have been used in precision medicine [25]. Studies that investigated policies addressing these “pairs” focused on oncology [26,27,28]. More recently, using companion diagnostics in the area of infectious diseases has been debated, with a view to optimising AMR stewardship policies [25,29]. In fact, even if not officially approved as companion diagnostics, several diagnostics for infectious diseases are used as companion diagnostics [29].

Policymakers are responsible for developing and implementing appropriate policies to achieve intended policy objectives [30]. For medicines, several policies (e.g., related to pricing, procurement and reimbursement (funding/coverage) have been introduced in European countries [31,32,33,34] that aim to ensure equitable and sustainable patient access to affordable medicines. Evaluations of the policies have identified strengths and limitations: For instance, pricing policies helped to contain prices and make medicines affordable to patients and health systems. Careful selection of medicines to be included in publicly funded reimbursement systems has also contributed to enhanced access. Demand-side measures targeting prescribers, pharmacists, and patients to build capacity and trust into generic medicines helped to free resources for innovative medicines while maintaining access to high-quality equivalent medicines. However, opaque pricing mechanisms around confidential discounts have led to greater information asymmetry between suppliers and public purchasers, and the widespread use of external (international) price referencing has proven to be one important factor of delayed market launches in countries of lower incomes [34,35,36,37,38,39,40].

However, little is known about pricing and funding policies, including the consideration of health technology assessments (HTA), for diagnostic tests in European countries. Even descriptive analyses of the national policy frameworks of POC diagnostics are missing for most countries.

To shed some light into this under-researched area, this study surveyed and comparatively analysed pricing and funding (reimbursement and remuneration) policies as well as HTA approaches for POC diagnostic tests applied for community-acquired acute respiratory tract infections (CA-ARTI diagnostics) in the outpatient sector across Europe. It aimed to identify specificities of the policies for these devices, as compared to other diagnostics and medical devices, in the European countries.

This mapping of current policies for CA-ARTI diagnostics was conducted with a view to informing research and policy making. Evidence from other countries allows learning lessons for implementation strategies in one’s own country. Moreover, it offers a basis for evaluating the impacts of the policies, and the findings of evaluations may support policymakers to strengthen the existing policy framework.

The study addressed the dearth of evidence regarding HTA, pricing and funding policies for POC CA-ARTI diagnostics in European countries. The findings point to a pricing and funding situation in several European countries in which price regulation is rarely applied for CA-ARTI diagnostics, incentives for health professionals to use these diagnostics are few, and HTA processes and outcomes are not well integrated in the decision-making processes.

## 2. Materials and Methods

### 2.1. Selection of Countries and Scope of Setting

To ensure broad coverage across Europe, the member states of the European Union (EU, 28 countries at the time of the survey); the European Free Trade Association (EFTA) countries Iceland, Norway and Switzerland; and Turkey were approached.

As the study concerned the community-acquired variant of ARTI, policies relating to use of POC diagnostics in outpatient care were investigated. The research was focused on POC tests conducted in general practices; tests which needed to be sent to laboratories were not considered. The hospital sector was not in the scope of the study.

### 2.2. Framework

Policies are defined as instruments, tools and approaches that allow policymakers (i.e., public payers, procurers and/or pricing authorities) to achieve defined policy objectives, such as fostering innovation, cost-containment or appropriate use of health technologies [41]; (commercial) strategies of private sector actors (e.g., suppliers) are not considered as policies and are not addressed in this paper.

**Figure 1:**
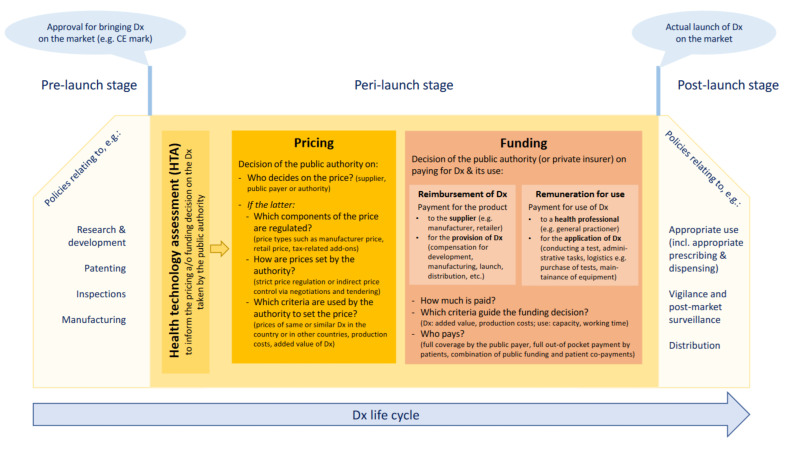
Framework for studied pricing and funding policies. Source: Authors. CE: Conformité Européenne/European Conformity; Dx: diagnostic(s).

To the best knowledge of the authors, no overarching framework for pricing and funding policies for medical devices, including diagnostic tests, exists, whereas generally accepted concepts and taxonomies have been developed for pharmaceuticals. For the purpose of this research, we developed a framework for the studied policies, presented in Figure 1. Based on the life-cycle approach for medicines that has been disseminated by the World Health Organization and related institutions [36,38], this framework takes into consideration the specificities of medical devices. In particular, it accounts for the fact that in addition to reimbursement of defined diagnostics (product-specific funding), health professionals may be remunerated for using procedures in which diagnostic tests are applied.

### 2.3. Data Collection and Validation

Information was collected through a qualitative survey of public authorities for pricing and reimbursement in European countries. This data collection method was chosen because a literature review conducted earlier by the authors had failed to identify sufficient information on country pricing and funding policies applied for diagnostic tests.

The questionnaire contained questions about the current use of HTA, pricing and funding policies applied for CA-ARTI diagnostics in outpatient care. The survey also explored whether or not, and how, the policies for CA-ARTI diagnostics differed from those implemented for other medical devices (see Supplementary File S1 for the sample questionnaire). The questionnaire was accompanied by an Informed Consent Form, and written informed consent was obtained from the respondents.

To ease the workload for participants, the questionnaire was pre-filled, where data was available. Inputs were mainly obtained from an unpublished study of the authors on pricing and funding policies for medical devices in general as well as a search of websites of relevant national public institutions in this field. Respondents were asked to provide up-to-date information as of the year 2020.

A draft questionnaire was piloted with two countries (Austria and Greece) in June 2020, and the rollout for all countries was launched in July 2020. Up to two personalised reminders were sent (September till November 2020). In January 2021, a summary of the findings was shared with all who have responded to the survey, and they were invited to check correctness of the presentation of their country.

Respondents of the survey included technical experts of national competent authorities responsible for pricing and reimbursement working on, or with an interest in, medical devices. They were members of the Pharmaceutical Pricing and Reimbursement Information (PPRI) Subgroup on Medical Devices. The PPRI network is a more than 15-year-old collaboration of competent authorities for pricing and reimbursement of medicines that included 52, mainly European, countries in 2020 [42]. To foster research and expert networking on medical devices, the PPRI Subgroup on Medical Devices (PPRI MD) was established in 2018 [43].

During the analysis of the data, respondents were contacted to clarify ambiguous answers and gain a better understanding of the information provided.

## 3. Results

### 3.1. Included Countries

**Figure 2:**
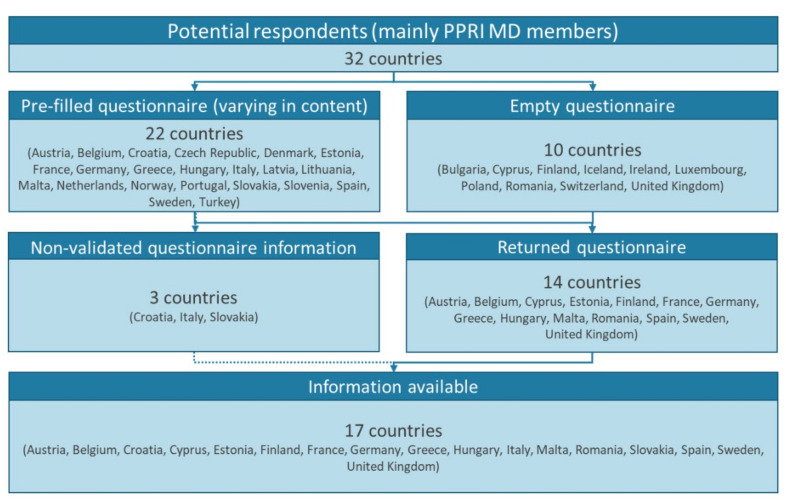
Study countries. MD: medical device(s); PPRI MD: Pharmaceutical Pricing and Reimbursement Information Subgroup on Medical Devices.

The study presents findings for 17 European countries. Thereof, national experts responded by providing information and validating pre-filled data in the questionnaire in 14 countries. Additionally, information was sufficiently available for three further countries and, though not validated, included in the results (Figure 2 and Supplementary File S2).

### 3.2. Use of HTA

In none of the 17 studied European countries were CA-ARTI diagnostics systematically assessed by an HTA as a basis for a pricing and funding decision. In several countries (e.g., Cyprus, Finland, Greece, Malta, Romania, Slovakia), the pricing and funding decision process did not include an HTA of any medical device. In some other countries (e.g., Austria, Belgium, Estonia, Spain), an HTA could be conducted for selected medical devices (products) or for procedures and methods in which devices were used. CA-ARTI diagnostics were not reported to be among the medical devices subject to an HTA. Countries reported that the HTAs were usually focused on new devices with higher price tags or high-risk classes. Studied dimensions tended to include clinical efficacy and safety; few countries (e.g., Belgium, Croatia) considered economic criteria in the assessment of medical devices. The HTAs were either conducted by regional HTA bodies (e.g., in Spain) or by national HTA bodies (e.g., Federaal Kenniscentrum voor de Gezondheidszorg, KCE, in Belgium; Haute Autorité de Santé, HAS, in France; Institut für Qualität und Wirtschaftlichkeit im Gesundheitswesen, IQWiG, in Germany).

### 3.3. Pricing Policies

In seven of the studied countries, the pricing authority set the price of some medical devices. The decision was based on the price of similar devices used in that country (internal price referencing) if available and applicable. For new medical devices subject to price regulation, a few countries (Belgium, France and Slovakia) considered the prices of the device in other countries (external price referencing), and France also took the expected value of the device into account when setting the price. With the exemption of Belgium, price regulation related only to the devices that were included in the outpatient positive list, i.e., those eligible for public funding. However, the reimbursement list did not include CA-ARTI diagnostics in any of the studied countries (Table 1).

Whereas price regulation describes a situation in which a public authority sets the price, its lack comprises free pricing (price of medical devices set by the supplier) or a negotiation or tendering process (thus, indirect price control through procurement). Studied countries reported frequent use of public procurement processes for CA-ARTI diagnostics.

**Figure 3:**
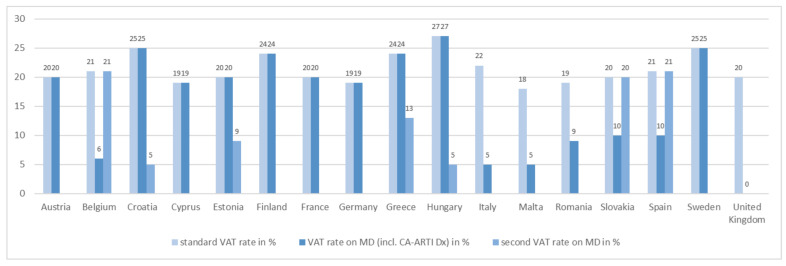
VAT rates for CA-ARTI diagnostics. CA-ARTI: community-acquired acute respiratory tract infections; Dx: diagnostic(s); MD: medical device(s); incl.: inclusive; VAT: value-added tax.

The final price is also determined by taxes. Apart from the United Kingdom, value-added tax was charged for CA-ARTI diagnostics and further medical devices in all study countries (Figure 3).

### 3.4. Funding Policies

A comparably high number of the studied countries reported that a POC CA-ARTI diagnostic test conducted in outpatient doctor’s practices was not covered by public funding, so patients had to pay for it (Table 2). In a few countries, these diagnostics were publicly funded, without or with co-payments charged from patients. Intra-country regional variations also existed (e.g., in Germany and Sweden).

Mechanisms for funding POC CA-ARTI diagnostics include the reimbursement of the POC diagnostic and the remuneration of the general practitioner (or any other outpatient health professional) for conducting the test in her/his practice. The latter is a funding policy that was used more commonly, whereas product-specific funding of the CA-ARTI diagnostics was rare, even in countries which maintained a reimbursement list (positive list) for medical devices eligible for public funding (Table 3).

## 4. Discussion

To the best knowledge of the authors, this study is the first cross-country survey of HTA, pricing and funding policies for CA-ARTI diagnostics. The lack of European comparative policy information for these diagnostics adds to a dearth of evidence on the respective policies for medical devices, including diagnostic tests, in most European countries. Studies tend to relate to high-risk medical devices in large markets, frequently used in the hospital sector [44,45,46,47,48]. The best available published evidence is grey literature, which provides, often in local language, information on policies for medical devices in a few large countries (e.g., France [49]). Thus, the data collection for several European countries, including smaller markets (such as Cyprus, Estonia or Malta), which tend to be under-researched, is an asset of this study.

Adding to limited implementation of pricing and funding policies, the lack of HTA processes for CA-ARTI diagnostics is a critical finding that requires further attention. This result adds to studies which identified a lack of methodological guidance for HTA on specific types of medical devices and shortcomings with evidence requirements [50,51].

As HTA is a tool to collate and assess evidence to support the decision on the inclusion of technologies into a publicly funded health system [52,53], its limited use for CA-ARTI diagnostics might be explained by the funding policies which governments in most studied countries apply for these devices. Most European countries do not provide product-specific funding for CA-ARTI diagnostics in outpatient use, and in such a situation an HTA of the diagnostic may not be considered of relevance.

HTA may also be used to inform pricing decisions. As the majority of the studied countries have no price regulation for medical devices, including CA-ARTI diagnostics, no need for an HTA may be seen. Use of HTA was in particular reported from countries which regulate the price of medical devices (but not for CA-ARTI diagnostics), such as France, Hungary, Spain and Sweden. This raises the question of whether or not the pricing and funding policy framework for medical devices has been sufficiently developed to consider the HTA outcomes appropriately. It is worth noting that existing pricing policies for medical devices tend to base the decision on the prices of similar devices in the same country and, to a lesser extent, on the price of the same device abroad. The value of a medical device, or a procedure in which a device is used, is not a routine decision criterion in a pricing process. Its application was solely identified for France—a country with an HTA body and a well-established and robust HTA methodology.

If we compare the identified policies to those applied for medicines, important differences can be observed. In the pharmaceutical system, internal price referencing, a commonly used policy for medical devices, is focused on off-patent medicines (generics and biosimilars), whereas further pricing policies apply for patent-protected medicines [31,35]. For new medicines, public authorities in many European countries first apply external price referencing (i.e., comparing to the price of the same medicine in other countries), frequently followed by negotiations with the manufacturers to achieve more affordable prices [54]. The latter may result in a so-called managed entry agreement, such as a risk-sharing, pay-for-performance or coverage-with-evidence scheme [55,56,57]. Value, expressed as the added therapeutic benefit compared to alternatives, including the ability of the medicine to address unmet medical (and societal) need, constitutes a key factor in determining the price of a new medicine, and HTA is needed to assess the value [58].

Further development of the pricing and funding processes for medical devices, with a view to synthesizing and analysing evidence in an HTA, will likely require a differentiated approach to account for the specificities of the different groups of medical devices, since the devices market is highly heterogenous. CA-ARTI diagnostics and POC tests in general may need more targeted pricing and funding policies that differ from those applied for other devices.

Our study findings allow making some high-level observations. It is known (e.g., from other health technologies) that pricing policies contribute to affordable and equitable prices [35] and that policymakers can implement them strategically to achieve defined policy objectives (e.g., in the area of medicines, to foster competition in the off-patent market and to benefit from efficiency gains due to the use of generic and biosimilar medicines [32,59]). From economic theory, high prices are, in principle, an incentive for suppliers; however, this is only the case if there is the ability to pay and willingness to pay from the purchasers’ side. Thus, for technologies with high price tags, free pricing combined with coverage (funding) by the public payers would be a major incentive granted to suppliers (an example, again from the pharmaceutical policy area, is Germany’s policy to fund medicines during the first twelve months at a price set by the company [60]). Even without public funding, free pricing by industry may serve as an incentive for suppliers to launch in cases of lower-priced technologies, whose cost can be borne by patients without financial hardship. An evaluation of a POC testing service in community pharmacies in Canada gave some indications that patients were willing to use this service despite being required to pay for it [61].

Before implementing any policy action, it is important to understand possible impacts of the supply-side and demand-side policies. Can specific pricing and funding policies incentivize launch and uptake of CA-ARTI diagnostics, or are, rather, measures targeting prescribers, pharmacists and patients required to address the reluctance towards these diagnostics? For instance, a study in England found that some high-prescribing doctors do not value the POC diagnostics as “clinical tools” [62]. This finding is interesting since POC tests have demonstrated high sensitivity and specificity rates [63,64]. It points to a need to include POC diagnostics in awareness-raising and capacity-building activities of AMR stewardship programmes.

This highlights that pricing and funding policies, which are the focus of this research, may not be sufficient and could be accompanied by further measures. As for other health technologies, a bundle of measures is needed (a so-called “mix-and-match’ package” as phrased by the European Joint Programming Initiative on Antimicrobial Resistance Transnational Working Group “Antimicrobial Resistance–Rapid Diagnostic Tests” [65]).

The study examined a specific group of medical devices. Medical devices comprise a broad range of products, and different pricing and funding policies may apply for different types of medical devices. Our survey findings relating to medical devices in general (serving as kind of “control group”) point to the following pattern in several European countries: some medical devices are considered as reimbursable and are included in an outpatient positive list for public funding (either complete coverage or including patient co-payments). For a few devices, an HTA is conducted to support the decision on inclusion in the positive list. Some similarities exist with regard to the policy framework applicable for medicines for outpatient use of European countries, where eligible medicines (e.g., given their added therapeutic value compared to alternatives or, in case of its lack, being offered at lower price) are funded by the public payers. Differences between medical devices and medicines concern the scope of technologies in the positive lists (e.g., a considerably lower number of devices included in reimbursement) and the relevance of HTA processes (e.g., for new medicines HTA is systematically conducted in several European countries [31], whereas HTAs conducted with the explicit aim to inform pricing and funding decisions of devices are rare). As another similarity between medicines and medical devices, prices tend to be regulated for those technologies that are eligible for reimbursement. From the medicine area, the rationale that policymakers aim to control the prices of the products they fund is known, and a similar approach for medical devices might be assumed. A major difference between medicines and medical devices concerns the funding mechanism: sometimes medical devices are not reimbursed on a product basis, but health professionals are remunerated for procedures in which they apply certain devices, such as CA-ARTI diagnostics.

Adding to these standard pricing and funding policies, additional policies, subsumed as new pricing models or novel payment schemes, have been developed, or are discussed, for some medicines, including vaccines. These developments concern, for instance, one-off treatments in rare diseases (e.g., Advanced Therapy Medicinal Products/ATMPs) and novel antibiotics [66,67,68]. Society has an interest (and need) that antibiotics with a lower probability of producing AMR be developed and marketed. Pricing and funding policies of novel antibiotics include specific funding schemes for these medicines (e.g., individual product-based reimbursement instead of bundled financing through diagnosis-related groups, which are usually applied in hospitals), exemptions from HTA and higher prices without proof of added therapeutic value [69,70,71]. In addition, new procurement mechanisms that de-link the reward for manufacturers from the volume and thus contribute to more appropriate use (so-called “Netflix” models) have been piloted for antibiotics in Sweden and the United Kingdom [72]. To steer the development of needed antibiotics, Advance Purchase Agreements (APA), which specify the target product profile, have been used. In the COVID-19 pandemic, the EU Member States used the APA policy in the joint procurement of the vaccines [39].

These examples concern advanced policies and may not immediately be transferable to diagnostics, for which standard policies are not yet always in place. However, one future approach could account for the specificity of POC CA-ARTI diagnostics that can be considered to serve as companion diagnostics. As such, a policy for encouraging the uptake of CA-ARTI diagnostics would benefit from a holistic approach that would include a joint policy for antibiotics and diagnostics. For instance, public funding of antibiotics would only be granted if the need had been demonstrated by a POC test. Different variants of implementation, accounting for a country’s policy characteristics, are possible (e.g., indicators on prescribing of antibiotics linked to use of CA-ARTI diagnostics, patient co-payments for prescribed antibiotics if their necessity had not been proven by a publicly funded test to incentivize patients to ask for the test). Ideally, these pricing and funding policies are combined with awareness campaigns for prescribers, pharmacists and patients.

The study does not come without limitations. Pricing and funding of medical devices, particularly of POC diagnostic tests, is an under-researched area, and this implies a lack of a theoretical basis. Thus, the authors first had to develop a framework for the analysis. While this work was guided by taxonomies used for medicines, it is acknowledged that there are some limitations regarding the transferability from the pharmaceutical to the medical devices sector.

It was a challenge to identify experts who were able to offer input and validate information. In three countries, information gained from literature and previous research could not be evaluated. Even public officials working on policies for medical devices were struggling to provide responses on specific policies targeting CA-ARTI diagnostics. While the authors aimed to mitigate any risks as far as possible (e.g., by intensive communication on unclear and ambiguous topics and answers), misunderstandings, including wrong responses by the country experts, are possible. Lack of expertise in the countries may also be attributable to the limited implementation of policies for these diagnostics, even in countries with HTA processes and pricing and funding policies for other devices. It is difficult to comment on policies if they are not in place; frequently, policies were implemented for other medical devices but not for POC CA-ARTI diagnostics. Thus, experts were knowledgeable regarding some other devices but not necessarily the diagnostics.

## 5. Conclusions

The study provides a comparative overview of pricing and funding policies for CA-ARTI diagnostics in European countries. The findings point to limited use of HTA in pricing and funding processes, low levels of price regulation, lack of public funding and few incentives for health professionals to use POC diagnostics. The study highlights a need for strengthening the policy framework, including in the areas of pricing and funding. Policies applied for other medical devices and for medicines (e.g., inclusion of products in a reimbursement list upon a rigorous HTA) may serve as a model. Drawing from knowledge of policies for medicines, a policy mix should be sought which accompanies pricing and funding policies for CA-ARTI diagnostics by demand-side measures targeting prescribers, pharmacists, and patients. In addition, as a rather novel approach, joint policies addressing both the use of the POC diagnostic test and antibiotic prescribing can be beneficial, as they account for the characteristics of CA-ARTI diagnostics as companion diagnostics.

This study is a starting point in the research on pricing and funding policies for POC CA-ARTI diagnostics. Based on its findings, further research can explore hindering and enabling factors of the policies identified in the study countries. The theoretical framework developed for POC diagnostic tests for the purpose of this study provides an important basis which can be optimised, with the view to extending it to other groups of medical devices. Further development of the analytical framework should consider the heterogeneity of the medical devices market, and any theory needs to be aligned with the respective group of medical devices investigated.

## Figures and Tables

**Table 1:** Price regulation for CA-ARTI diagnostics.

Country	Price Regulation for	
	CA-ARTI Diagnostics	Further Medical Devices
Austria	No	No
Belgium	No	Only for hearing aids
Croatia	No	No
Cyprus	No	No
Estonia	No	No
Finland	No answer available	No answer available
France	No	For MD included in the reimbursement list
Germany	No	No
Greece	No	For MD included in the reimbursement list
Hungary	No	For MD included in the reimbursement list
Italy	No	No
Malta	No	No
Romania	No	No
Slovakia	No	For MD included in the reimbursement list
Spain	No	For MD included in the reimbursement list
Sweden	No	For MD included in the reimbursement list
United Kingdom	No	No

CA-ARTI: community-acquired acute respiratory tract infections; MD: medical device(s).

**Table 2:** Funding sources for CA-ARTI diagnostics.

Reimbursement Status of POC Diagnostic Tests for CA-ARTI	Country
Public payer pays, full coverage of CA-ARTI Dx classified as reimbursable	Cyprus, Estonia, Finland, Romania, Slovakia
Public payer pays for CA-ARTI Dx classified as reimbursable, supplemented by patient co-payments	Austria ^1^, France ^2^
Patients pay out-of-pocket for CA-ARTI Dx	Belgium, Croatia, Germany ^3^, Greece, Hungary, Italy ^4^, Malta ^4^, Spain ^4^, Sweden ^5^, United Kingdom

^1^ Depending on the social health insurance fund (Austria). ^2^ In practice, co-payment is covered by a “mututelle” (complementary health insurance) which most French citizens have. ^3^ Public funding for tests sent to laboratories (their use is encouraged before antibiotic prescribing), not for POC CA-ARTI diagnostics, with the exception of one region (Sachsen-Anhalt) where use of POC CA-ARTI diagnostics is funded by social health insurance. ^4^ Italy, Malta and Spain: Up to the general practitioner to decide whether or not to charge the patient for having the POC CA-ARTI test done; Spain: while out-of-pocket payments by patients are charged on a routine basis for CA-ARTI diagnostics, some POC CA-ARTI diagnostics may be included in outpatient reimbursement list for some strategic rationale. ^5^ Co-payments vary between the 21 Swedish regions. CA-ARTI: community-acquired acute respiratory tract infections; Dx: Diagnostic(s); POC: point-of-care.

**Table 3:** Funding policies for CA-ARTI diagnostics.

Country	Reimbursement for the Device		Remuneration for the Service	
	POC CA-ARTI Dx	Further MD	POC CA-ARTI Dx	Further MD
Austria	Yes	Yes	Yes	Yes
Belgium	No	Yes	No	Yes
Croatia	No	Yes	No information	No information
Cyprus	Yes	Yes	Yes	Yes
Estonia	Yes	Yes	Yes	Yes
Finland	Yes	Yes	No information	No information
France	No	Yes	Yes	Yes
Germany	No	Yes	No	Yes
Greece	No	Yes	No	Yes
Hungary	No	Yes	No	Yes
Italy	No	No	No	No
Malta	No	No	No	No
Romania	Yes	Yes	No	Yes
Slovakia	Yes	Yes	No	Yes
Spain	Yes, partly	Yes	No	No
Sweden	No	No	No	Yes
United Kingdom	No	Yes	No	Yes

CA-ARTI: community-acquired acute respiratory tract infections; Dx: Diagnostic(s); MD: medical device(s); POC: point-of-care.

## Data Availability

The data presented in this study are available in the article here.

## Supplementary Materials

The following are available online at https://www.mdpi.com/article/10.3390/antibiotics11080987/s1, Supplementary File S1: Empty questionnaire; Supplementary File S2: List of responding institutions in the study countries.
