# Supplementary material for: Assessing, Pricing and Funding Point-of-Care Diagnostic Tests for Community-Acquired Acute Respiratory Tract Infections–Overview of Policies Applied in 17 European Countries
Source: Antibiotics (Basel). 2022 Jul 22;11(8):987. doi: 10.3390/antibiotics11080987 (PMC9331460; doi:10.3390/antibiotics11080987)
Supplement: Supplementary file 1 [file antibiotics-11-00987-s001.zip › antibiotics-1820248-supplementary.pdf]

Supplementary File S1: Empty questionnaire.

**Scope of survey - Products**	
Are **fast diagnostic tests** for (community-acquired acute) respiratory tract infections (RTI or CA-ARTI) **used in the outpatient sector** in [*country name*]? (e.g. CRP test)?<br>Note: Besides "normal" diagnostic tests for RTI or CA-ARTI, this question addresses also new tests that generate results within minutes. E.g.:<br>• Serological tests (e.g. C-Reactive Protein (CRP), Procalcitonin (PCT))<br>• Real-time/nested multiplex RT-PCR,<br>• Urine antigen test<br>• Mass spectromy<br>• Multiplex microarray competitive DNA hybridization,<br>• Isothermal RT-helicase-dependent amplification (I),<br>• Loop-mediated isothermal DNA amplification (LAMP),<br>• (Isothermal) nucleic acid amplification /polymerase chain reaction	
If yes, **which** of these **specific diagnostic tests** are already **in use** in the outpatient sector?<br>Note: see examples of products of different suppliers which also exemplifies the different types (e.g. CRP, PCR, etc.) in Annex 1	
If yes, how is the decision made to use these specific tests (e.g. practitioner, guidelines, etc.)?	
**Health Technology Assessment (HTA)**	
What is your **national HTA institution** / department in charge of assessing **medical devices (MD) and diagnostics (IVD)**?	
Are **funding (reimbursement) and/or pricing** decisions for fast diagnostic tests for RTI or CA-ARTI based on **prior HTA**?<br><br>If yes, which **under which conditions**?	
Briefly describe the **HTA process for fast diagnostic tests for RTI or CA-ARTI**?<br><br>*Kindly elaborate*:<br>• Which dimensions (effectiveness, safety, cost-effectiveness) are covered?<br>• Which criteria / indicators are applied?	
Do **HTA processes** for **fast diagnostic tests for RTI or CA-ARTI differ from those for other MD**, incl. diagnostic tests in general? What are the differences?<br>Note: To assess patient-relevant outcomes, consequences (sensitivity, specificity, safety, etc.) of the test and also any subsequent therapies can be taken into account. This can be done through direct comparison of the therapeutic consequences of the new test with those of the reference test or by "linked evidence" (linking studies on diagnostic accuracy to studies on therapeutic effectiveness).	
**Reimbursement (includes funding and/or coverage)**	
Are **fast diagnostic tests for RTI or CA-ARTI** (e.g. CRP) **reimbursed by public payers in the outpatient sector** in [*country name*]?<br><br>If yes, could you **provide an estimate** of the number of reimbursed fast diagnostic tests for RTI or CA-ARTI (or the share of fast vs. "normal" diagnostic tests for RTI or CA-ARTI)?<br><br>Note: The focus lies on point-of-care-testing and tests that generate results within minutes.	
**How** are **fast diagnostic tests for RTI or CA-ARTI reimbursed** in the outpatient setting?<br><br>Note: possible mechanisms include: fee-for-service, pay-for-performance, lump sum or product-based funding<br><br>Are there **specific incentives** regarding the reimbursement of fast diagnostic tests for RTI or CA-ARTI used in the outpatient setting?	
Are the **reimbursement** mechanisms for **fast diagnostic tests for RTI or CA-ARTI different from those for other MD**, including diagnostic tests in general?<br>If yes, **how do the mechanisms differ**?	
Kindly explain the (decision) procedures regarding the **inclusion of fast diagnostic tests for RTI or CA-ARTI into reimbursement**?	
Are the **reimbursement decision** procedures for fast **diagnostic tests for RTI or CA-ARTI different from those for other MD**, including diagnostic tests in general?<br>If yes, **how** do the reimbursement **decisions differ**?	

Are there patient **co-payments** charged **for fast diagnostic tests for RTI or CA-ARTI**? If yes, **which** are these co-payments?	
Do patient **co-payments** charged for fast **diagnostic tests for RTI or CA-ARTI differ from those charged for other MD**, including diagnostic tests in general?<br>If yes, **how** do the **co-payments differ**?	
Is there a **discussion** ongoing that **reimbursement** of **fast diagnostic tests for RTI or CA-ARTI** should be (further) **incentivised**?<br>Note: e.g. higher reimbursement rate/amount, alternative reimbursement schemes to incentivise the uptake of diagnostic tests (which are faster, have a higher sensitivity/ specificity, test for a high number of viruses/bacteria)<br>If yes, what is the content of this discussion?	
If yes, who are the **involved stakeholders**?<br>Please provide some references, e.g. from media, websites (in national language is fine)	
**Pricing**	
Are **prices** of **fast diagnostic tests for RTI or CA-ARTI** used in the outpatient sector **regulated** by law?<br><br>If yes, **how** are the **prices regulated** and for which types of fast diagnostic tests for RTI or CA-ARTI?	
Does **price regulation for fast diagnostic tests** for RTI or CA-ARTI **differ from** price control **diagnostic tests or other MD** in general?<br>If yes, **how does price regulation differ**?	
Which price type of **fast diagnostic tests for RTI or CA-ARTI** used in the outpatient sector is price-regulated?<br>Note: price types are ex-factory price, wholesale price (pharmacy purchasing price), pharmacy retail price?<br>Are there **differences to other MD**, incl. diagnostic tests in general?	
Which **additional pricing policies** are applied for **fast diagnostic tests for RTI or CA-ARTI** used in the outpatient sector?	
None (there is free pricing for medical devices)	
Do **pricing policies and applied criteria differ** for fast diagnostic tests for RTI or CA-ARTI from those applied for other MD, including diagnostic tests in general?<br>If yes, **how**? Are higher prices granted? Any (price) incentives?	
Kindly inform whether, or not, the following **pricing policies** are applied for **fast diagnostic tests for RTI or CA-ARTI** used in the outpatient sector**: external price referencing, internal price referencing, value-based pricing, tendering and cost-plus pricing**<br>Note: for definitions see the glossary at the end of the survey	
Are there any **differences** related to these **pricing policies** between their application for fast diagnostic tests for **RTI or CA-ARTI and for other MD**, including diagnostic tests in general?	
Are wholesale and pharmacy mark-ups regulated for **fast diagnostic tests for RTI or CA-ARTI**?<br>(How) are community pharmacies remunerated for supplying fast diagnostic tests for RTI or CA-ARTI?	
If yes, how does **wholesale and pharmacy mark-up regulation for fast diagnostic tests for RTI or CA-ARTI differ from** those applied for other **MD**, including diagnostic tests in general?	
Are **sales taxes** (e.g. VAT) applied on **fast diagnostic tests for RTI or CA-ARTI**, and if yes, at which price type?	
If yes, how **sales taxes for fast diagnostic tests for RTI or CA-ARTI differ from** those applied for other **MD**, including diagnostic tests in general?	
Is there a **discussion** ongoing on **incentivizing pricing/prices** of **fast diagnostic tests for RTI or CA-ARTI**?<br>Note: e.g. higher prices for defined diagnostic tests (which are faster, have a higher sensitivity/ specificity, test for a high number of viruses/bacteria)<br>If yes, what is the content of this discussion?	
If yes, who are the **involved stakeholders**?<br>If possible, please provide **some references**, e.g. from media, websites (in national language is fine)	
**Anything to add**	
Any **literature / documents/ evaluation studies** that we should consult?	
Feel free to add any **further comments** reg. HTA, reimbursement or pricing for fast diagnostic tests for RTI or CA-ARTI.	

THANK YOU VERY MUCH FOR YOUR SUPPORT!

Supplementary File S2: List of responding institutions in the study countries.

Country	Institution
Austria	Sozialversicherungsanstalt der Selbständigen (SVS) / Social insurance institution for the self-employed; Österreichische Gesundheitskasse (ÖGK) / Austrian Social Health Insurance Fund
Belgium	Institut national d'assurance maladie-invalidité (RIZIV) / National Institute of health and disability insurance (NIHDI)
Cyprus	Οργανισμός Ασφάλισης Υγείας / Health Insurance Organisation (HIO)
Estonia	Eesti Haigekassa / Estonian Health Insurance Fund (EHIF)
Finland	Kansallinen HTA-koordinaatioyksikkö / Finnish Coordinating Center for Health Technology Assessment (FinCCHTA)
France	Comité Economique des Produits de Santé / Health Products Pricing Committee (CEPS)
Germany	GKV-Spitzenverband / National Association of Statutory Health Insurance Funds
Greece	ΕΥΡΩΠΑΪΚΗ ΚΑΡΤΑ ΑΣΦΑΛΙΣΗΣ / National Organisation for the Provision of Health Services (EOPYY)
Hungary	Nemzeti Egészségbiztosítási Alapkezelő / National Institute of Health Insurance Fund Management (NEAK)
Malta	Directorate for Pharmaceutical Affairs, Ministry of Health
Romania	Directia politica medicamentului a dispozitivelor și tehnologiilor medicale, Ministerul Sănătății / Medicines, Medical Devices and Technologies Policy Directorate, Ministry of Health
Spain	Cartera Básica de Servicios del SNS y Farmacia, Ministerio de Sanidad, Consumo y Bienestar Social / Directorate General for NHS Common Services Portfolio and Pharmacy, Ministry of Health
Sweden	Tandvårds- och läkemedelsförmånsverket / Dental and Pharmaceutical Benefits Agency (TLV)
United Kingdom	Pricing and Costing Department, NHS England and Improvement

A pre-filled questionnaire was sent to public authorities in Croatia, Italy and Slovakia, with a request for validation. However, despite reminders the information was not validated.
